# Enhancing digital workflows for removable partial dentures: A novel diagnostic surveyor and designer

**DOI:** 10.1111/jopr.14059

**Published:** 2025-04-18

**Authors:** Ahmed Mahrous, David L. Botsko, Tamer El‐Gendy, Amira Elgreatly

**Affiliations:** ^1^ Arizona School of Dentistry and Oral Health A. T. Still University Mesa Arizona USA; ^2^ Private practice Davenport Iowa USA

## Abstract

Removable partial dentures (RPDs) are a valuable treatment option in prosthodontics, often selected due to patient‐specific limitations such as financial constraints, inadequate bone support, or medical contraindications to other prosthetic solutions. Despite the advantages of digital dentistry, such as the efficiency and accuracy of various workflows, the adoption of fully digital RPD workflows has lagged due to the absence of accessible diagnostic surveying and design tools for digital models. This article introduces the AiDENTAL RPD Surveyor and Designer, a lightweight, browser‐based software solution that simplifies digital surveying, streamlines design, and facilitates the export of diagnostic scans. By addressing these needs in the current digital workflow, the system aims to enhance the efficiency, accuracy, and convenience of RPD fabrication. A step‐by‐step description of the process highlights its potential to improve clinical outcomes and reintegrate traditional framework‐based RPDs into mainstream practice.

Removable partial dentures (RPDs) have long been a cornerstone in the prosthetic treatment options available to prosthodontists and restorative dentists. While alternative prostheses may offer better comfort and function, RPDs remain the treatment of choice in certain situations; RPDs frequently remain the treatment of choice in scenarios involving financial limitations, inadequate bone for implant restorations, or medical contraindications to complex prosthodontic solutions. These considerations underscore the enduring significance of RPDs in delivering optimal patient care. ^1^, ^2^, ^3^

Traditional RPD workflows involve multiple steps, often requiring significant time and manual effort at each stage.^2^ The emergence of digital dentistry has transformed many prosthodontic procedures by introducing speed, precision, and convenience. One of the most significant digital transformations was the development of digital impressions as an alternative to traditional diagnostic and final impressions which introduced many advantages including increased accuracy, time efficiency, and patient comfort.^4^ However, digital workflows for RPDs have not kept pace and have largely remained traditional.^5^ This gap in digital RPD workflows stems from the absence of a feasible digital alternative to traditional diagnostic surveying and design tools.

Diagnostic surveying and design are crucial for determining the optimal path of insertion, identifying undercuts, and planning retention, ensuring the stability and functionality of the prosthesis. While digital tools have been developed to assist in the surveying and design of RPDs, many are primarily intended for use in dental laboratory settings during the fabrication of the removable partial denture framework. These tools often lack the functionality required for comprehensive diagnostic surveying, such as tripoding, saving specific tilts, marking undercut areas for use, or effectively communicating design plans to a dental laboratory.^6^, ^7^, ^8^

Digital laboratory work authorizations allow seamless transmission of digital files between the clinician and the lab, eliminating the need for manual documentation. However, in the field of RPDs, design drawings are still done manually, forcing the clinician to draw the design on paper and then inconveniently digitize or “scan” the paper design to include a digital laboratory work authorization. ^9^ This manual process introduces the potential for errors and inefficiencies, hindering the adoption of digital workflows in removable partial denture fabrication.

These limitations discourage the adoption of digital workflows for RPDs, leading many dental practices to opt for less technique‐sensitive alternatives, such as flexible resin RPDs, which are traditionally used as interim solutions. ^10^

This article introduces a digital alternative to traditional diagnostic surveying and design, aiming to bridge the current gap in the RPD digital workflow. The AiDENTAL RPD Surveyor and Designer is a tool designed to provide an accessible and time‐efficient digital solution compared to the currently available traditional and digital options for diagnostic surveying.

The AiDENTAL Surveyor and Designer is a web‐based application that can be accessed through any internet browser. It allows users to upload STL files of digital impressions, which can then be tilted to the desired path of insertion and surveyed in a manner similar to traditional surveying on a physical diagnostic cast. The software automates the identification of edentulous segments, abutment teeth, and favorable retentive undercuts at the selected path of insertion. This data is then transferred to the integrated RPD design module, which generates an algorithm‐driven custom RPD design based on established design principles.

Furthermore, the software enables convenient digital communication with the dental laboratory. It allows users to mark areas on the digital model, save the generated tilt and survey lines, and export the digital design. These features can be seamlessly incorporated into a digital laboratory work authorization along with the final impression. The entire process takes between 2 and 5 min and is designed to streamline chairside digital surveying and design, reducing the number of steps, appointments, and procedures needed to transition from the diagnostic phase of RPD to the final impression and laboratory authorization.

By integrating digital surveying, automated design generation, and seamless laboratory communication, the AiDENTAL RPD Surveyor and Designer offers a comprehensive solution to streamline the RPD workflow. To demonstrate the practical application of this system, the following section provides a step‐by‐step guide outlining its use in clinical practice. This technique showcases how digital surveying and design can be efficiently incorporated into the RPD workflow, enhancing precision, reducing manual effort, and optimizing case planning.

## TECHNIQUE

**FIGURE 1:**
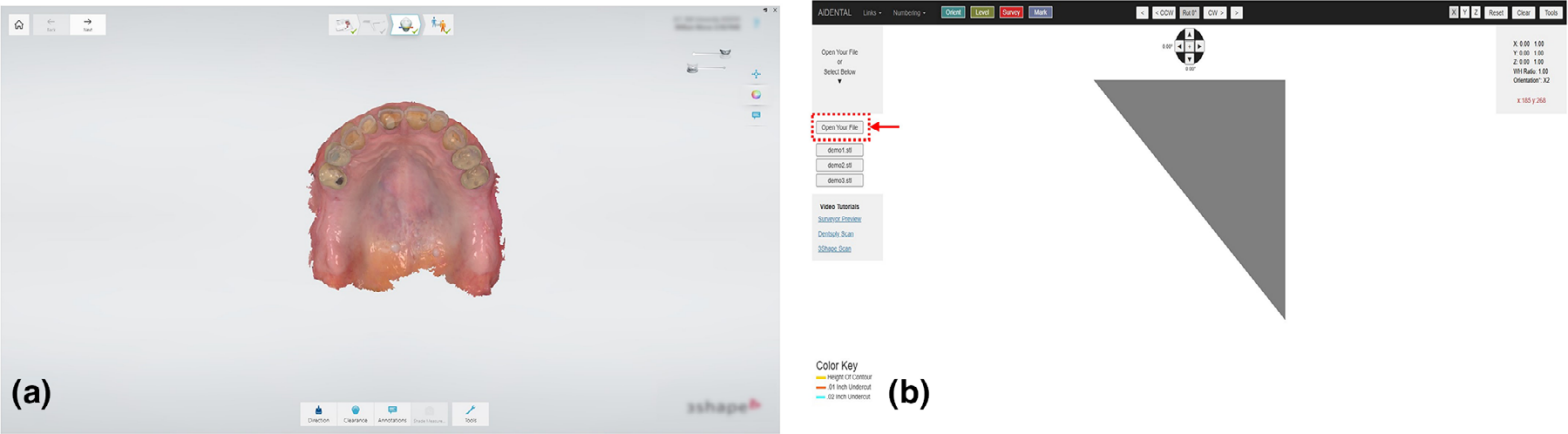
(a) An intraoral diagnostic scan completed on an intraoral scanner and exported into an STL file. (b) On the AiDental Surveyor page, the model can be uploaded using the indicated button.

**FIGURE 2:**
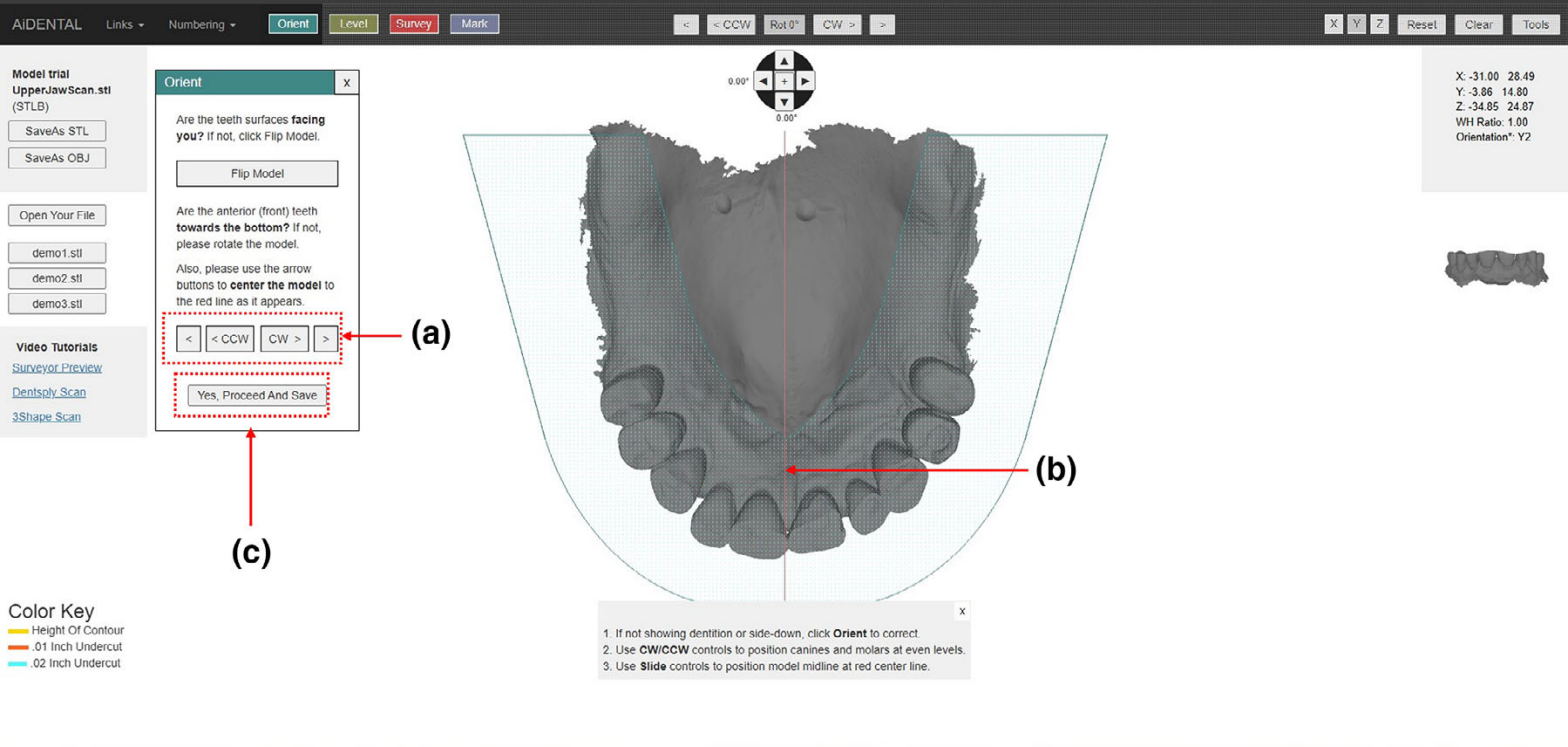
The model is uploaded into the AiDental Surveyor and is shown on the “Orientation” page. The Surveyor attempts to automatically orient the model, however, some manual orientation is sometimes needed depending on the shape and quality of the STL. (a) Manual orientation is done by using the directional buttons on the orient panel. (b) The model should be positioned so that the mid‐line corresponds to the redline on the screen and the teeth reside within the shaded arch form. (c) Once the position is satisfactory the user should press the indicated button to move on to the next step.

**FIGURE 3:**
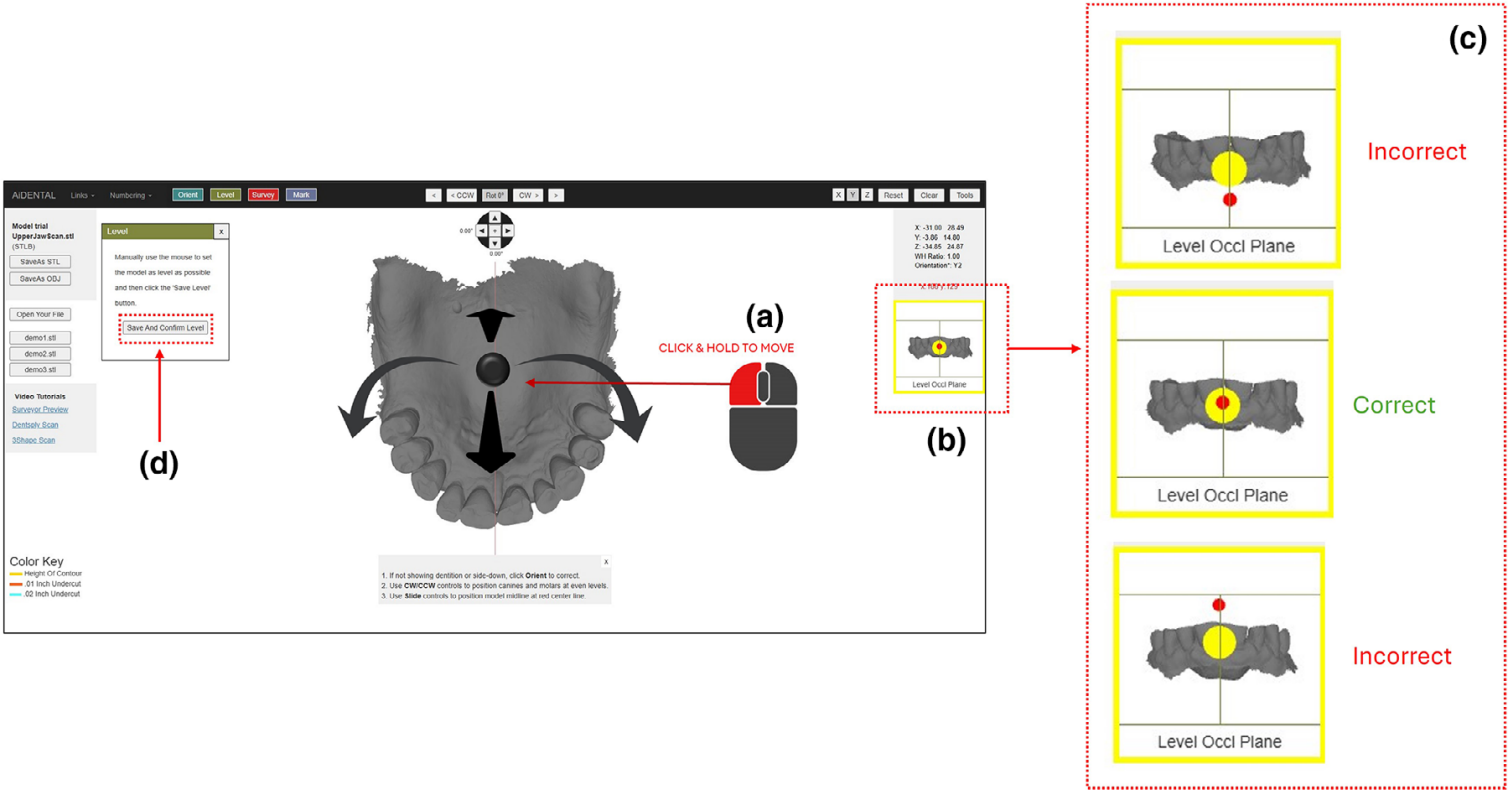
The second step of the surveying is the “Leveling” which aims to level the occlusal plane so that it is parallel to the horizon. (a) The user can use the left button of the mouse to click on the center of the model to tilt the model. (b) The front view panel is used to assist in leveling. The left‐right leveling is achieved by visually confirming that the occlusal plane is parallel to the horizontal lines, and the anterior‐posterior leveling is achieved by confirming that the red dot is positioned in the center of the yellow circle as shown in the middle figure. (d) Once the leveling is completed the user can select the indicated button to proceed to the next step.

**FIGURE 4:**
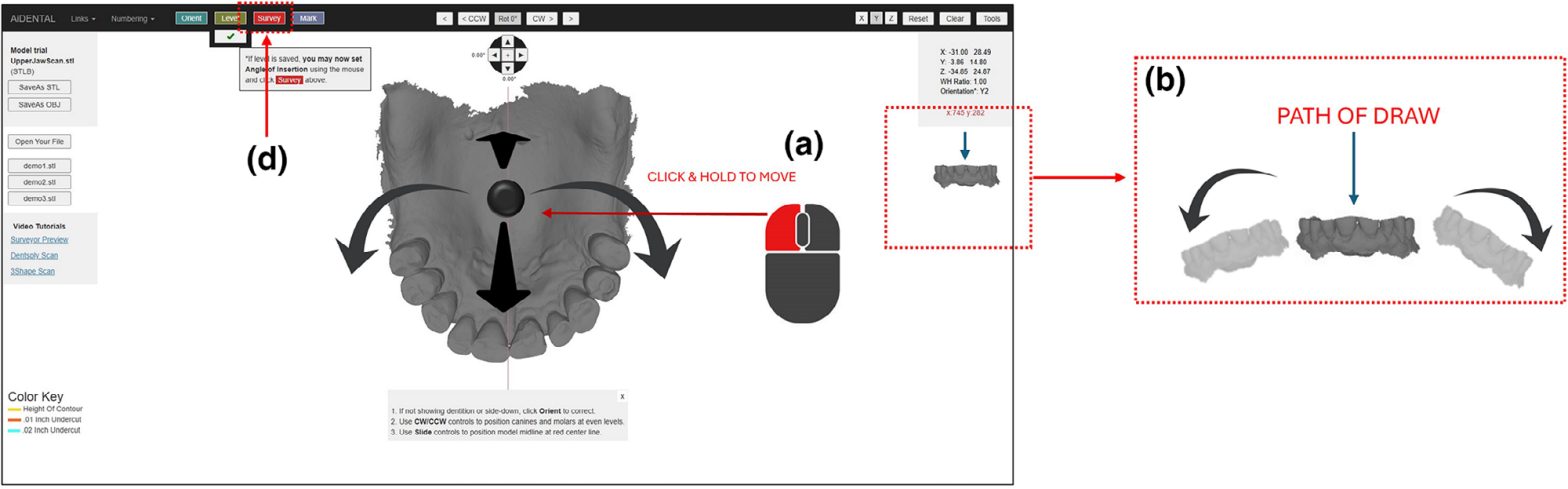
The third step is to set the path of draw and survey the model. (a) The user can use the left button of the mouse to click on the center of the model to tilt the model. (b) This step is similar to the previous step, however, this step changes the angle at which the teeth will be surveyed relative to the path of draw. (d) Once the path of draw is set the user can proceed to survey by selecting the indicated button.

**FIGURE 5:**
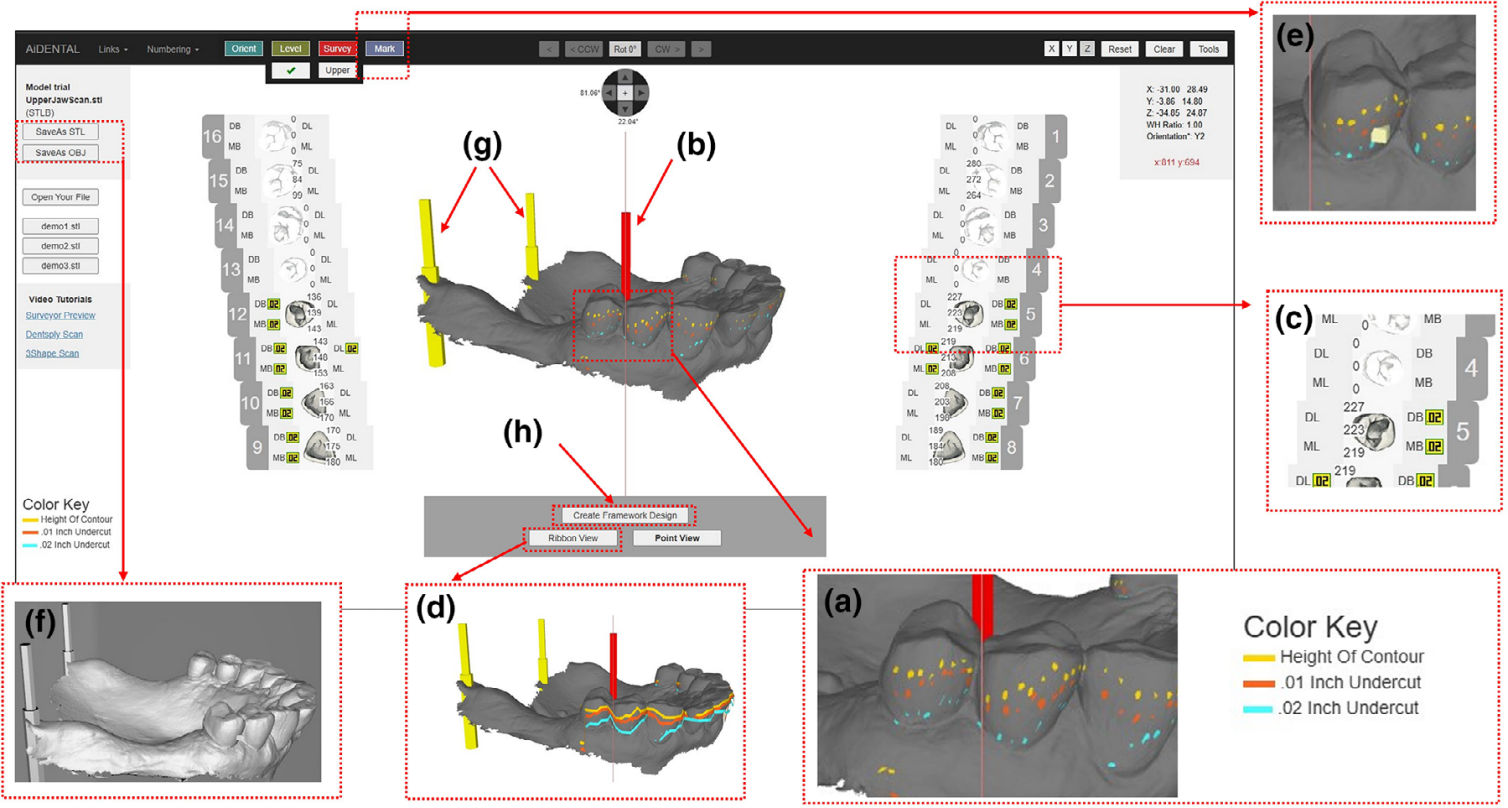
The surveyed model is shown. (a) The height of the contour, 0.01” undercut and 0.02” undercut locations are marked on the individual vertices of the model. (b) The selected path of draw is depicted with the red rod. If a different path of draw is needed or the model needs to be tilted differently the user can “Reset” the model to re‐tilt the model to a new path of draw. (c) The surveyor will automatically detect missing and present teeth during surveying and will attach the corresponding undercuts to their respective tooth surfaces. However, inaccuracies might exist due to non‐ideal tooth position or scanning artifacts in which case manual input can made by the user by clicking and unclicking the present and missing teeth as well as manually matching the listed undercuts to the ones on the model. (d) The ribbon view can be used to visualize the undercut areas as a continuous line, which may assist in visualizing undercut locations in low‐resolution areas of the model. (e) Areas of interest can be marked by selecting the “ Mark” tool and clicking on areas of the model. This will leave a small rectangular shape on the model which can be used to make note of certain areas of interest for the treatment plan or to facilitate communication with the dental laboratory. (f) The model can be exported to a new STL, which will retain the marked areas as well as two (g) “Ney Surveyor adaptors” that can be used to mount a printed model to a Ney surveyor at the same tilt used during virtual surveying. The exported STL also retains all height of contour data and will show once more if re‐uploaded onto the AiDental Surveyor. (h) After a satisfactory survey result is achieved, the user can then select the indicated button to proceed to the AiDental Designer and design a custom RPD design. RPD, removable partial denture.

**FIGURE 6:**
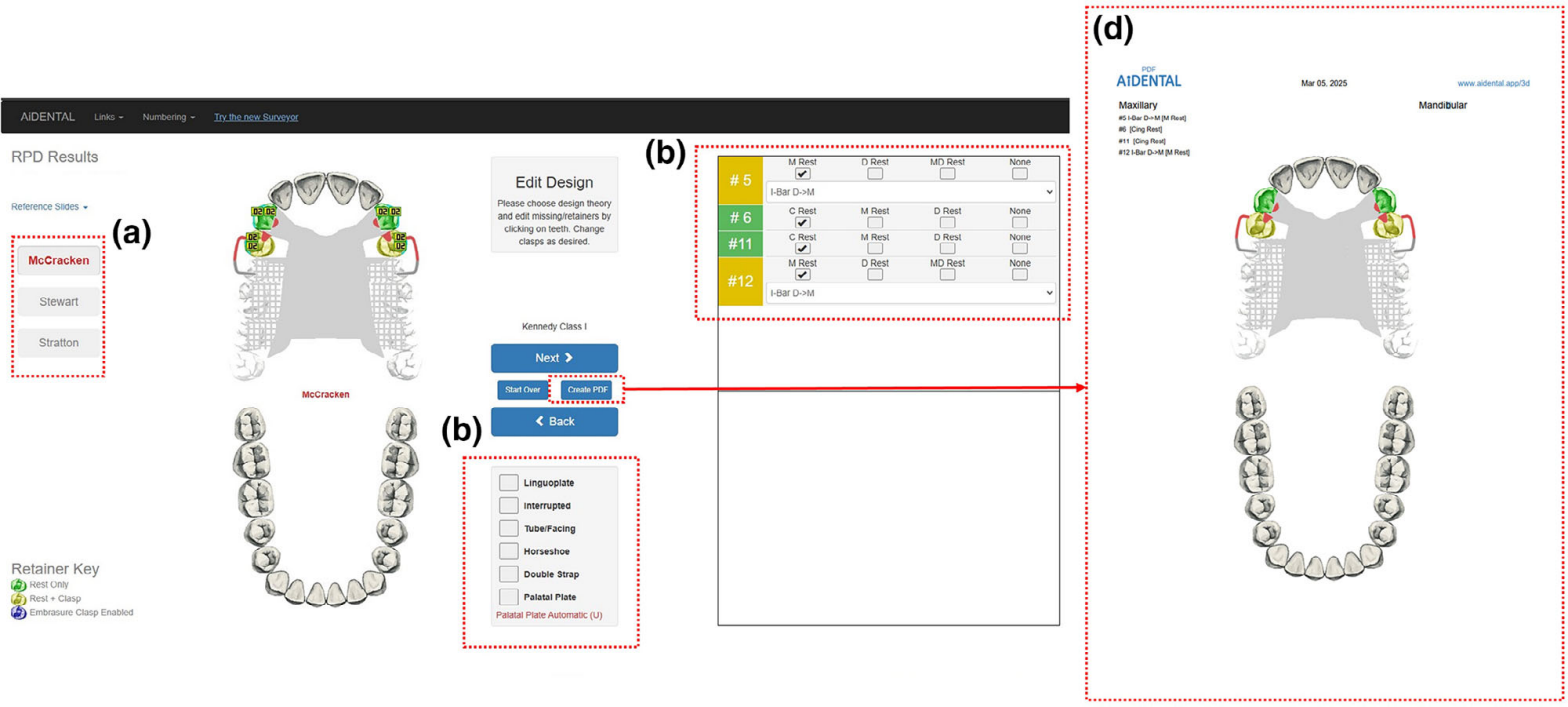
The algorithm‐based design is generated in the AiDental Designer component. (a) The design is generated based on the selected RPD philosophy. (b) The design can be customized to the practitioner's preference by choosing different types and locations for clasps, rests, and major connector designs. All changes will be reflected on the diagram instantaneously. (d) Once the design is completed the practitioner can export the design to a PDF containing the design as well as any selected clasp information for convenient record‐keeping and laboratory communication. RPD, removable partial denture.

**FIGURE 7:**
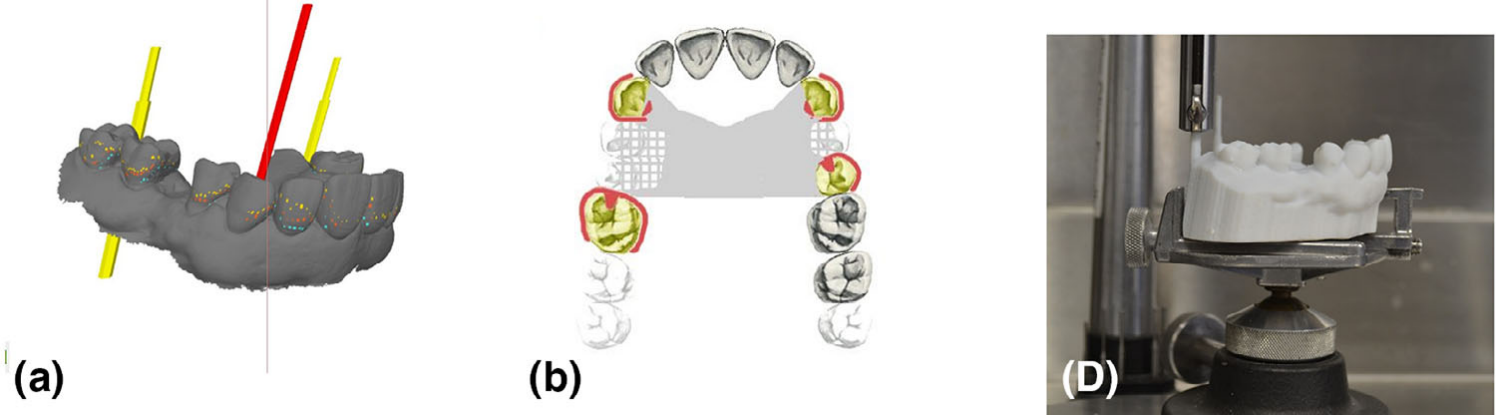
(a) Tripoding is achieved on the exported model because when the model is uploaded into AiDental again the model will show all the relevant height of contour data at the previously selected path of draw, as well as any marked areas indicated by the practitioner. (b) The exported digital design will complement the tripoded model and will facilitate convenient laboratory communication. (d) If physical mounting is needed, the provided adapters will allow mounting onto a “ Ney Surveyor” onto the previously selected path of draw.

1. Acquire a diagnostic intraoral scan using an intraoral scanner and export the file in STL format (Figure 1a). The STL file may be uploaded to the AiDENTAL Surveyor (Figure 1b).
2. Orient the digital model on the “Orientation” page. The software attempts automatic orientation; however, manual adjustments may be needed depending on the scan quality (e.g., the presence of artifacts or incomplete data) (Figure 2a). The model should be aligned so that the midline corresponds to the red reference line, and the teeth fit within the shaded arch form (Figure 2b). Adjustments are made using directional buttons, and once a satisfactory orientation is achieved, proceed to the next step (Figure 2c).
3. Level the occlusal plane to ensure parallelism with the horizontal reference lines. This step parallels the leveling process traditionally performed on a physical surveyor. The model may be adjusted by clicking on its center and tilting it to achieve proper leveling (Figure 3a). The front‐view panel aids in confirming left‐right leveling by ensuring the occlusal plane aligns with the horizontal lines. The anterior‐posterior leveling is adjusted using a red dot and yellow circle as reference markers (Figure 3b). Once properly leveled, advance to the next step (Figure 3d).
4. Establish the optimal path of draw to ensure proper distribution of undercuts for clasp retention. The model may be adjusted by clicking and tilting it, similar to the previous step, to modify the angle at which the teeth will be surveyed relative to the path of draw (Figure 4a,b). Once the desired path of draw is set, proceed to the survey by selecting the indicated button (Figure 4d).
5. Analyze the survey results and mark critical areas. Upon completion of the survey, the software automatically identifies and marks the height of the contour, 0.01‐inch undercuts, and 0.02‐inch undercuts on the model (Figure 5a). The chosen path of drawing is visualized with a red reference rod (Figure 5b). If modifications are needed, the model may be reset for re‐orientation. The software detects missing and present teeth and assigns undercuts; accordingly, however, manual adjustments can be made if inaccuracies exist due to tooth malposition or scanning artifacts (Figure 5c). The ribbon view provides a continuous visualization of undercut locations, aiding assessment (Figure 5d). Areas of interest may also be marked, remaining visible on the model after exporting to assist in treatment planning and laboratory communication (Figure 5e). The final surveyed model may be exported as an STL file, retaining all contour data and survey markings, and it includes Ney Surveyor adapters for physical mounting at the selected tilt (Figure 5f,g). Once the survey is finalized, proceed to the design phase (Figure 5h).
6. Generate an RPD framework using the AiDENTAL Designer component. An algorithm‐based RPD framework is generated based on the selected design philosophy (Figure 6a). The framework may be modified by selecting clasp types, rest locations, and major connector designs, with real‐time visualization of the changes (Figure 6b). Once finalized, the design may be exported as a PDF containing the framework design and clasp details for record‐keeping and laboratory communication (Figure 6d).
7. Retain survey data for consistency. The exported model maintains all survey data, allowing the previous surveying session to be recreated when re‐uploaded into AiDENTAL. The height of the contour, undercut locations, marks, and path of draw remain unchanged (Figure 7a). The digital RPD design complements the tripoded model, facilitating efficient laboratory communication (Figure 7b). If physical mounting is required, the provided Ney Surveyor adapters enable proper positioning at the previously selected path of draw (Figure 7d).

## DISCUSSION

The described workflow eliminates many of the limitations associated with traditional and hybrid analog‐digital RPD fabrication. By allowing diagnostic models to be surveyed and designed digitally, the need for costly laboratory software or manual surveying equipment is reduced. Additionally, the integration of digitally marked undercuts enhances the accuracy and consistency of RPD framework fabrication in the laboratory. In addition to its clinical applications, AiDENTAL includes an educational component, the Game, which provides an interactive, game‐based learning environment for dental students. This tool offers consistent, calibrated feedback on RPD design exercises, helping students refine their skills and deepen their understanding of RPD design principles in a practical and engaging manner. The Game component complements the Designer and Surveyor tools, making AiDENTAL a comprehensive platform for both clinical and educational use. The AiDENTAL Designer has demonstrated effectiveness in improving dental students’ understanding of RPD design by providing calibrated feedback on their work.^11^ The addition of the AiDENTAL Surveyor to the platform now addresses key barriers to adopting digital workflows in RPD fabrication, particularly the lack of digital surveyors designed specifically for diagnostic casts and the effective communication of surveying data to dental laboratories. By offering clinicians a practical tool for digital surveying and design, the system facilitates the transition toward fully digital workflows.

The advantages of this system include improved time and cost efficiency, as digital surveying eliminates the need for printing or casting diagnostic models, reducing both material costs and appointment times. Additionally, digital tools enhance accuracy by minimizing human error in surveying and ensuring precise documentation of undercuts. The ability to export designs and diagnostic scans in digital formats further streamlines communication with dental laboratories, reducing turnaround times and improving case coordination.

Despite these advantages, the system may have limitations in cases involving severely compromised or malposed teeth or complex occlusal relationships, where manual adjustments may still be necessary. Further studies are needed to assess its performance in such cases and refine the workflow accordingly.

It is important to note that all designs and recommendations provided by AiDENTAL should be considered as guidance rather than definitive treatment plans. Practitioners should always reference their clinical experience and judgment when finalizing RPD designs to ensure patient‐specific needs are met.

## SUMMARY

The AiDENTAL RPD Surveyor and Designer represents a significant advancement in the digital workflow for RPD fabrication. By bridging the gap in digital diagnostic surveying and design, the system enhances efficiency, accuracy, and communication while maintaining the clinical utility of framework‐based RPDs. As digital dentistry continues to evolve, integrating such tools into daily practice and education will support the broader adoption of streamlined, technology‐driven workflows in removable prosthodontics.

## CONFLICT OF INTEREST STATEMENT

Dr. Ahmed Mahrous and Dr. David L. Botsko are consultants and developers of the AiDental Application and Surveyor described in this manuscript. The other authors declare no conflicts of interest.
